# Assessment of bacterial diversity of *Rhipicephalus microplus* ticks from two livestock agroecosystems in Antioquia, Colombia

**DOI:** 10.1371/journal.pone.0234005

**Published:** 2020-07-01

**Authors:** Juan A. Segura, Juan P. Isaza, Luz E. Botero, Juan F. Alzate, Lina A. Gutiérrez

**Affiliations:** 1 Grupo Biología de Sistemas, Escuela de Ciencias de la Salud, Facultad de Medicina, Universidad Pontificia Bolivariana, Medellín, Antioquia, Colombia; 2 Departamento de Microbiología y Parasitología, Facultad de Medicina, Centro Nacional de Secuenciación Genómica - CNSG, Sede de Investigación Universitaria - SIU, Universidad de Antioquia, Medellín, Antioquia, Colombia; University of California San Diego, UNITED STATES

## Abstract

*Rhipicephalus microplus* is recognized as a tick species highly prevalent in cattle, with a wide pantropical distribution that seems to continue spreading geographically. However, its role as a biological vector has been scarcely studied in the livestock context. In this study, a 16S rRNA next-generation sequencing analysis was used to determine bacterial diversity in salivary glands and gut of *R*. *microplus* from two contrasting livestock agroecosystems in Antioquia, Colombia. Both the culture-independent approach (CI) and the culture-dependent (CD) approach were complementarily adopted in this study. A total of 341 unique OTUs were assigned, the richness showed to be higher in the Northern than in the Middle Magdalena region, and a high diversity was found at the phylum and genus levels in the samples obtained. With the CI approach, Proteobacteria, Bacteroidetes, Firmicutes, and Actinobacteria were the most common phylum of bacteria regardless of the organ, or geographic origin of the specimens analyzed. While the relative abundance of bacteria at a phylum level with the CD approach varied between analyzed samples, the data obtained suggest that a high diversity of species of bacteria occurs in *R*. *microplus* from both livestock agroecosystems. Bacterial genera such as *Anaplasma*, *Coxiella*, and *Ehrlichia*, recognized for their implications in tick-borne diseases, were also detected, together with endosymbionts such as *Lysinibacillus*, previously reported as a potential tool for biological control. This information is useful to deepen the knowledge about microbial diversity regarding the relations between endosymbionts and pathogens and could facilitate the future development of epidemiological surveillance in livestock systems.

## Introduction

Tick-borne diseases (TBD) are a public health problem in the world [1]. Ticks harbor a great diversity of microorganisms, some of which are pathogenic to humans and other mammals [2]. Ticks are obligate hematophagous ectoparasites associated with the transmission of viruses, bacteria, and protozoa, which affect both human and animal health [3,4]. *R*. *microplus*, considered as a relevant vector of tick-borne diseases in livestock agroecosystems around the world [5,6], is acknowledged as the most predominant cattle tick species. Other wild and domestic animals are recognized as part of their life cycle [7], and its geographical range keeps expanding.

During ticks’ blood-feeding process, the salivary glands and midgut are the primary organs for pathogens acquisition. However, transmission can vary depending on each pathogen and on aspects as the capacity of adaptation to the different environmental conditions that permit the migration from to the midgut to salivary glands or ovaries and the adapt to physiological and behavioral characteristics on the tick, such as blood-feeding, digestion, molting [8,9], and immune response [10]. *R*. *microplus* bacteriome was first reported in 2011, in South Texas, after using non-crop-based molecular approaches (Roche/454 sequencing platform) to evaluate DNA from whole ticks, gut, and ovaries, where bacteria such as *Wolbachia*, *Coxiella*, and *Borrelia* were detected. Besides, this study suggested that the changes in bacterial diversity observed in *R*. *microplus* could be influenced by its geographic distribution [5]. Another referent is a survey carried out in China, which compares independent and dependent culture methods to identify intestinal bacteria associated with *R*. *microplus* ticks in goats and bovines. The results indicated that the microbial diversity of the tick depends on its host and study site; also, several species, not previously reported in the area, were detected, such as *Coxiella* sp., *Ehrlichia* sp., *Rickettsia peacockii*, *Staphylococcus*, *Xanthomonas*, *Pseudomonas* and *Orphnebius* sp. [11]. Recently, also in China, whole specimens and saliva were examined to identify bacteria in partially and fully engorged *R*. *microplus* ticks. After employing Illumina HiSeq technology, the greatest richness of bacteria was detected in partially engorged ticks with a total of 111 OTUs, where Proteobacteria, Firmicutes, Actinobacteria were the predominant phylum, among others of the *Acinetobacter*, *Rickettsia*, *Escherichia* and *Coxiella* genera [12]. These antecedents show that changes in bacterial diversity in *R*. *microplus* depend on its geographic distribution, blood-feeding, and host. Both culture-dependent and culture-independent approaches were used to describe microbial diversity in ticks, detecting important species not previously reported. These studies served as our basis to explore bacterial diversity in the midgut and salivary glands of *R*. *microplus*, using similar approaches Colombian livestock context, which had not yet been explored.

In Colombia, livestock farming is a countrywide economic activity. The largest cattle population is raised in the department of Antioquia, where the Uraba, Northern, and Middle Magdalena regions stand out as the most significant producers at a department level [13]. Although *R*. *microplus* is present in these regions [14], its role as a biological vector of tick-borne diseases is not well understood yet. Considering the representativeness of the North and Middle Magdalena regions in the livestock sector of Antioquia, this study was aimed at assessing the bacterial diversity in salivary glands and gut of *R*. *microplus* female ticks collected in such regions. In Colombia, this is the first study providing information about bacterial diversity of *R*. *microplus* using 16S rRNA NGS- next-generation sequencing with the culture-independent and culture-dependent approaches. This work could be helpful to evaluate the risks of tick-borne diseases for cattle and humans in these areas, and as a basis to develop control strategies.

## Materials and methods

### Ethical approval

This study was approved by the Health Research Ethics Committee of the Universidad Pontificia Bolivariana (Record No. 5 of April 24, 2017). It was also granted an environmental license issued by the Colombian Government, through the National Environmental Licensing Authority (*Autoridad Nacional de Licencias Ambientales*-ANLA, Resolution ANLA 744 of July 26, 2016).

### Dataset and deposition

The 16S rRNA data from this study were available through the NCBI BioProject ID PRJNA525289. Also, the mitochondrial DNA 16S rRNA fragment (accession numbers: MN650725-MN650729) and the 18S rRNA amplified from *R*. *microplus* was deposited in GenBank (accession numbers: MN650730-MN650734). Some voucher specimens of *R*. *microplus* were deposited in the biological collection of the Alexander von Humboldt Biological Research Institute -IAvH (codes: IAvH-I-2951 to IAvH-I-2954). The dataset (https://doi.org/10.15472/hen46i; https://doi.org/10.15472/ncal7j) was uploaded to the SiB Colombia’s data portal (https://sibcolombia.net/).

### Sampling sites

In the Northern region, animals grazed in predominantly Kikuyu grass surrounding pastures (*Pennisetum clandestinum*). The main breeds were Holstein, Jersey, and Jersey x Holstein cattle (*Bos taurus*). Farms were located between 2298 and 2621 meters above sea level.

In the middle Magdalena region, the main breeds were Gyr, Jersey, and *Bos indicus* x *Bos taurus* cattle grazed in the surrounding jungle/forests, with predominantly Brachiaria (*Brachiaria* sp.) and Jaragua grass (*Hyparrhenia rufa*) pastures. Farms were between 182 and 497 meters above sea level.

**Fig 1:**
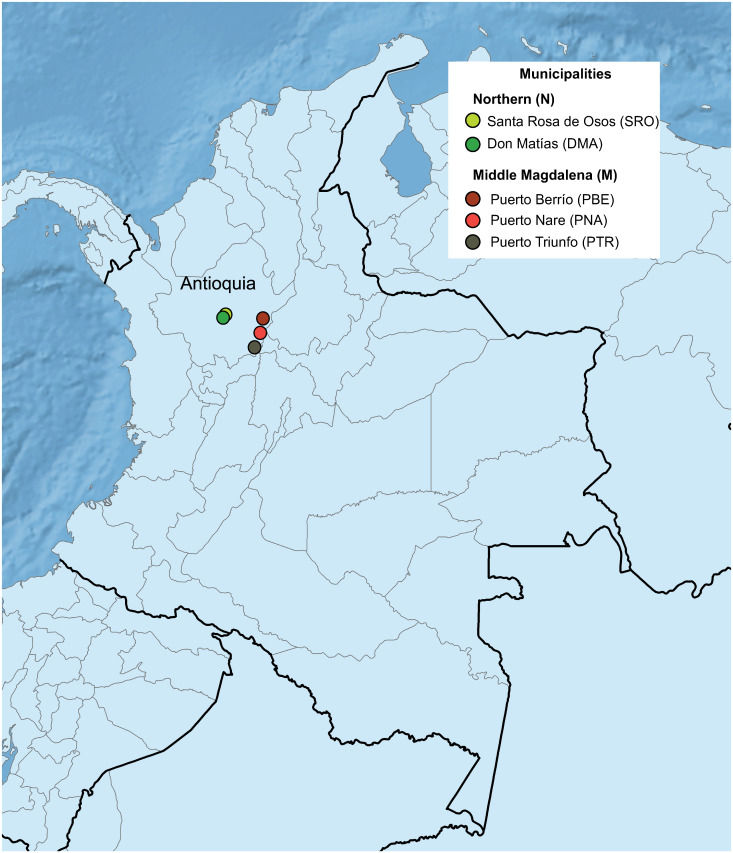
Geographic location of the municipalities included in this study. Dots represent geographical localization of the cattle farms included in this study within each of the five municipalities in the Department of Antioquia, Colombia. Information on geographical coordinates was represented using the ArcGIS^®^ 10.2 software Copyright© 1999–2013 Esri Inc. https://www.esri.com/en-us/arcgis/about-arcgis/overview Layers were extracted from Natural Earth (public domain): http://www.naturalearthdata.com/.

All farms were in the department of Antioquia, and their longitude and latitude coordinates were acquired using a global positioning system (GPS Trimble^®^ Juno^®^ 3B, Westminster, USA). The study was conducted in five sites selected from different agroecosystems areas. The geographical distribution of the study areas is presented in Fig 1.

### Sample collection and *R*. *microplus* identification

A total of 25 partially-fed female ticks (larger than 3.5 mm) were collected from 15 cattle between August and September 2017, following a protocol for direct collection of ticks from each bovine [15] and transported alive in sterile tubes. Each specimen was analyzed to confirm the growth stage, sex, and taxonomic status as *R*. *microplus*, based on morphological keys [16,17]. The microdissection of each type of organ, salivary glands or gut, was performed with a sterile scalpel blade (N°. 11), as previously described [18,19]. Using a stereomicroscope needle (Leica EZ4, Heerbrugg, Switzerland), the dorsal cuticle flap was lifted, and each organ was removed with fine forceps and. Five female ticks from each farm were dissected, and each salivary gland, gut, or exoskeleton obtained were grouped in pools by organ (S1 Table).

Genomic DNA was extracted from all partially-fed female ticks carcass material (Exoskeleton-material) of five arthropods pooled by the municipality to obtain molecular confirmation of their taxonomic status as *R*. *microplus*. This process was completed by using the mammalian tissue genomic DNA Purification Protocol from the GeneJET^™^ Genomic DNA Purification Kit (Thermo Scientific^™^, CA, USA). During to microdissection and DNA extraction process, ticks were manipulated under sterile conditions in a Class II biological safety cabinet (BSC) using cycles of ultraviolet (UV) light between uses to avoid the contamination.

Two different molecular markers were amplified following separate PCR protocols. The fragment mitochondrial DNA 16S rRNA was amplified using 16S-F (5’–TTAAATTGCTGTRGTATT-3’) and 16S-R1 (5’- CCGGTCTGAACTCASAWC-3’) primers [20], whereas the fragment 18S rRNA was amplified using usingNS3 (5’-GCAAGTCTGGTGCCAGCAGCC-3’) and NS4 (5’-CCGGTCTGAACTCASAWC-3’) [21] primers. DNA extraction and PCR master mix preparation and amplification were performed in separate rooms to avoid contamination. The amplification protocols previously proposed [20,21] were optimized using BIOLASE^™^ DNA Polymerase (Bioline, London, UK) following manufacturer instructions. This optimization resulted in annealing temperatures of 50°C and 60°C for mitochondrial DNA 16S rRNA and 18S rRNA primers, respectively. Blank controls (nuclease-free water) were included during the PCR, and PCR products were verified through agarose gel electrophoresis. Bidirectional sequencing (Macrogen Corp., MD, USA) was performed to obtain partial sequences of each molecular marker. Reference sequences of 18S rRNA and mitochondrial DNA 16S rRNA of Ixodidae ticks were downloaded from NCBI to conduct the molecular phylogenetic analysis, which included multiple sequence alignments with ClustalW 2.1 using Geneious Prime^®^ 2019.0.4 (https://www.geneious.com). A molecular evolution model was selected using the Bayesian criterion to select and to conduct a molecular phylogenetic analysis by the Maximum Likelihood (ML) method using MEGAX 10.0.5 [22]. The dendrogram was estimated by a heuristic search and a Bootstrap resampling with 1000 pseudo replicas (S1 Fig).

### Bacterial diversity assessment of *R*. *microplus*

Each pool of salivary glands (n = 5) or gut (n = 5) was homogenized and divided into two aliquots, one for the culture-independent (CI) approach and the other for the culture-dependent (CD) approach.

### Culture-independent (CI) approach

Each pool of salivary glands or gut was immediately placed in 2 ml sterile microcentrifuge tubes containing 180 μl buffer lysis, and 20 μl Proteinase K. Genomic DNA was extracted using PureLink^®^ Genomic DNA Mini Kit (Thermo Fisher Scientific, USA) following the manufacturer instructions. The DNA quality was checked by spectrophotometry in a Nanodrop^®^ 2000 (Thermo Fisher Scientific, USA).

PCR amplification was achieved using Bakt_341F (CCTACGGGNGGCWGCAG) and Bakt_805R (GACTACHVGGGTATCTAATCC) universal primers while targeting the V3 through V4 regions of the 16S rRNA gene [23]. PCR products were processed for library preparation with Nextera XT library preparation kit, PhiX control DNA, and sequenced with paired-end 2x250 reads on the MiSeq System (Illumina, USA) at Macrogen Inc. (Seoul, Rep. of Korea).

### Culture-dependent (CD) approach

A second aliquot sample of each pool of salivary glands or gut was resuspended in vials containing 200 ul of sterile 0.9% NaCl and homogenized by vortexing to achieve organ disruption and allowing the greatest release of bacteria; then, each sample was brought to a final volume of 1000 μl and vortexing again. Subsequently, 10 μl of the suspension were directly seeded in each different aerobic culture media (blood agar, chocolate agar, Mueller Hinton agar, Hecktoen agar, and SS agar). Such volume was chosen to obtain independent colonies in the culture media. Finally, the Petri dishes were incubated at 35 °C for 48 hours.

After incubation for 48 hours, the bacteria growth as colonies on the different solid media used for the culture of each organ (intestine or salivary glands) was verified. After this, washing the surface of the solid media with a final volume of 50 ml of sterile 0.9% NaCl, ensuring all the colonies were recovered [24]. Finally, the material obtained was centrifuged at 16000 RPM, and the pellet was processed for DNA extraction, PCR amplification, and sequencing targeting the V3 through V4 regions of the 16S rRNA gene, following the same culture-independent protocol, as explained in the previous section.

### Sequence processing and data analysis

Raw sequences were analyzed with Mothur v.1.39 [25], according to the MiSeq Standard Operating procedure -SOP [26]. Briefly, merged reads were filtered to a maximum length of 465 nt without ambiguous bases. Chimeras were removed with the VSEARCH algorithm [27]. OTUs with 97% of identity were built with the Opti clustering method included in Mothur v.1.39. Then, they were classified using the Ribosomal Database Project Trainset v16 MicrobiomeAnalyst [28]. Data were filtered to remove low count OTUs (parameters: minimum count 2; prevalence in samples: 10%). These were scaled using the cumulative sum scaling algorithm. Afterward, MicrobiomeAnalyst [28] web server was employed to calculate diversity indexes and core microbiome. For the former, regions were compared in terms of evenness (Shannon and Simpsons) and richness (observed OTUs). Methodological approaches, organs, and differences were tested using a T-test. For the latter, the following parameters were included: taxonomic level (genus), sample prevalence (20%), and relative abundance (0.01). Linear discriminant analysis (LDA) effective size [29] was performed to identify biomarkers through Galaxy web application [30] with the following parameters: Kruskal-Wallis test between classes: 0.05; Wilcoxon test between subclasses: 0.05 and Threshold on the logarithmic LDA score: 2. On the other hand, beta diversity was defined as the dissimilarity between communities, estimated using Principal coordinate Analysis (PcoA) ordination plot employing the Bray-Curtis dissimilarity index to evaluate diversity between each organ and methodological approaches, Permutational Multivariate Analysis of Variance (PERMANOVA) was conducted to test the association of microbial composition with covariates of interest in this study. It is important to mention that it could have possible background noise in this data, due to the absence of blank extraction controls during the DNA extraction and bioinformatics workflows.

### Molecular phylogeny analysis of selected bacteria genera: *Anaplasma*, *Ehrlichia* and *Coxiella*

Phylogenetic analyses by Bayesian inference were performed with the sequences of the OTUs detected and the reference sequences. Partial 16S rRNA nucleotide sequences of *Anaplasma* sp. and *Ehrlichia* sp. were retrieved from Genbank database (AF069758, AF318021, AB013008, AB013009, M73227, U96436, AF147752, AF416764, CDGH01000025, JX629805, AB723707, AB723708, AF318946, KM206273, KP314237, AB588977, AB211164, AF309866, AF309867, AF309865, AF318945, AB196475, AB211163, AF156784, AF286699, AB196720, AB196721). In the case of *Coxiella* sp. (AY939824, CP007541, KP994811, KP994812, KP994833, KP994836, JQ480824, KP994844, KU892220, KC170757, KC170760, KP994770, KP994768, KP994769, AY342037, CP018005, KP994823, KP994824, KP994780). Sequences were aligned using Muscle v5, and ambiguously aligned regions were removed with Gblocks v0.91b. For *Ehrlichia* sp. and *Anaplasma* sp. phylogeny, *Wolbachia* endosymbiont of *Drosophila mauritiana* (U17060) was used as the outgroup, while for *Coxiella* phylogeny, *Legionella anisa* (AJ969020) and *L*. *pneumophila* (AF129523) were used as outgroups. Phylogenetic analyses were conducted using the Bayesian Inference method implemented in MrBayes v3.2 with the General Time Reversible (GTR) substitution model with two parallel runs, and two million MCMC generations.

## Results and discussion

This study describes an initial exploration of the bacterial diversity of the tick species *R*. *microplus* in salivary glands and gut. Samples were collected in the Northern and Middle Magdalena, two ecologically contrasting livestock regions of Antioquia, Colombia. It also describes the bacteria identified using a direct approach with the massive sequencing of the V3 through V4 regions of the 16S rRNA gene versus the data obtained in samples subjected to a culture-dependent method.

### Ticks identification

The specimens analyzed here were identified as *R*. *microplus*, based on morphological characteristics confirmed by molecular and phylogenetic analyses inferred from mitochondrial DNA 16S rRNA (S1 Fig). This result confirmed the *R*. *microplus* identity of the specimens from Antioquia included in this work. Besides, it showed the close link between the specimens analyzed in this work and the clade B [31], usually restricted to *R*. *microplus* ticks found in America, Africa, Malaysia, and Japan [32,33] (S1 Fig). Regarding the phylogenetic analysis based on the 18S rRNA gene, partial sequences were grouped at the genus level as *Rhipicephalus*.

### Bacterial identification using 16S rRNA sequencing

As S1 Table shows, there were differences between the raw reads data of the sequenced libraries obtained with the culture-independent (CI) approach and the culture-dependent (CD) approach. The total number of raw reads was 1,929,897, combining the data of both CD and CI. After applying quality filters, 636,764 reads were assigned to 9,223 raw OTUs (Operational Taxonomic Units), out of which 8,024 were unique OTUs considering 97% of identity. Finally, from 1,237 filtered OTUs of both CD and CI, 341 OTUs remained after the removal of low-count OTUs. Filtered OTUs ranged from 11 to 142 per sample. The appendix includes a rarefaction curve (S2 Fig). CI approach exhibited a higher richness based on the observed OTUs (S3 Fig).

### Bacterial alpha diversity

Shannon’s and Simpson’s indices were estimated to evaluate diversity between organs, regions, and culture-independent and culture-dependent methodological approaches implemented in this study (S4 Fig). Only statistical differences were found between CI and CD approaches at the phylum level (p < 0.001) and genus level (p < 0.001, p = 0.019 respectively) (S4 Fig).

Shannon’s and Simpson’s indices at the phylum and genus level showed a lower diversity in the CD samples than in the CI samples (S4 Fig). Considering that the data obtained at the phylum level and genus level did not show differences between the organs, it was decided that further analyses would consider the totality of OTUs identified at the genus category regardless of the tick’s organ analyzed.

**Fig 2:**
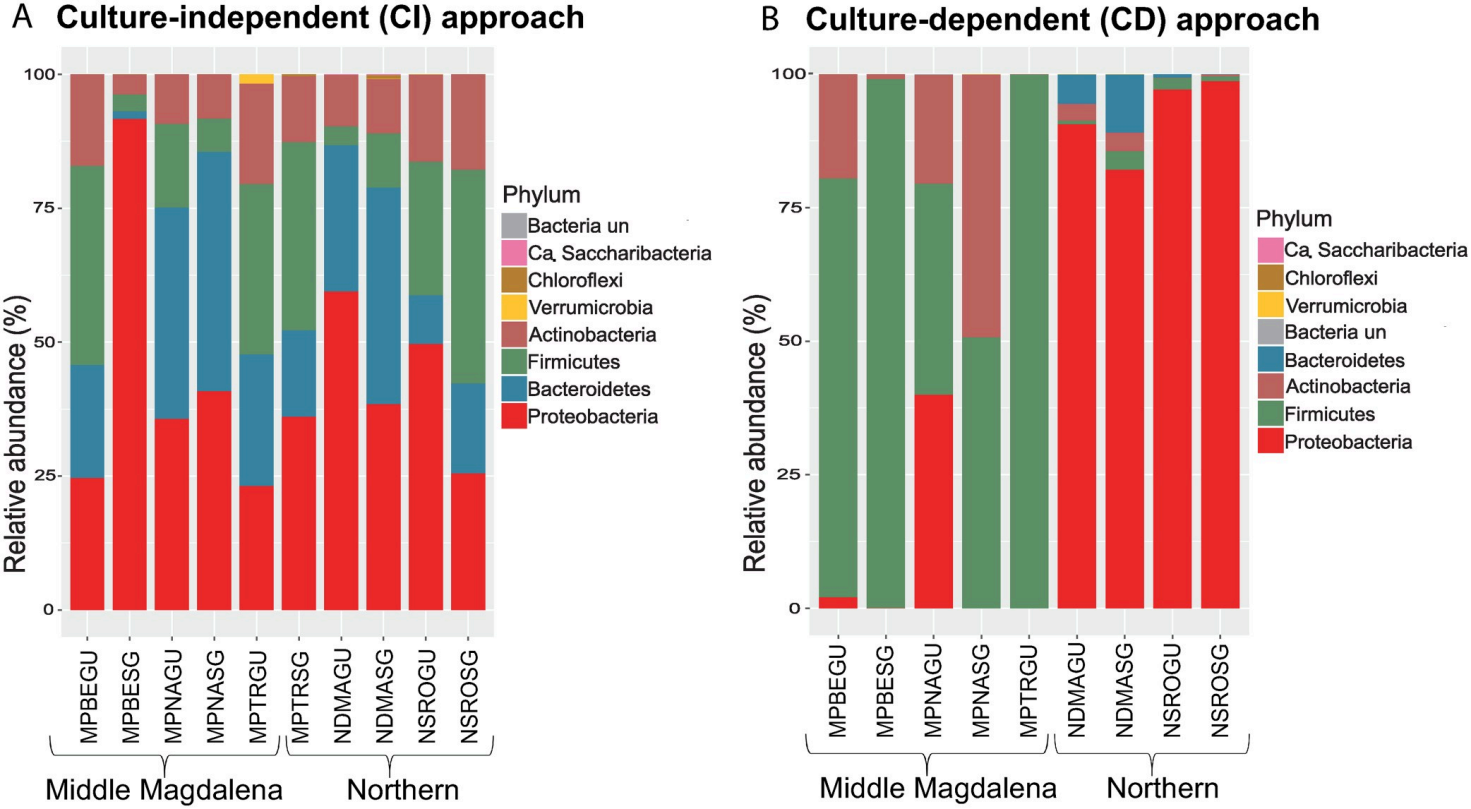
Relative abundance of bacteria at the phylum level. (A) Samples obtained with the culture-independent (CI) approach, (B) culture-dependent (CD) approach. Data are presented in proportion (%) to all sequences classified at this taxonomy level. Key: N: Northern region, M: Middle Magdalena region; PBE: Puerto Berrío; PNA: Puerto Nare; PTR: Puerto Triunfo; DMA: Don Matías; SRO: Santa Rosa de Osos; GU: Gut; SG: Salivary glands; un: unclassified; Ca: Candidatus.

Regardless of the organ or geographic origin of the specimens, the four most abundant phyla detected with the CI approach were Proteobacteria, Bacteroidetes, Firmicutes, and Actinobacteria. These added to approximately 95% of the classified reads (Fig 2A), while the relative abundance of bacteria at the phylum level with the CD approach varied between the samples analyzed. In the Northern region, a dominance of Proteobacteria was detected, whereas, in the Middle Magdalena region, Firmicutes was the most abundant phylum (Fig 2B). The differences in the bacterial diversity profiles to Northern and Middle Magdalena region were found. These variations can also depend on the host, as some authors have reported for goats and cattle [11], and lizards and mice [34].

Our findings are partially in agreement with a previous study on saliva or whole-body samples obtained from partially or fully engorged adult *R*. *microplus* females, in which Proteobacteria, Firmicutes, and Actinobacteria were also the predominant phyla; nevertheless, a relative abundance of the phylum Proteobacteria [12] higher than 75% was found.

In total, 112 bacterial genera were detected. Statistically significant differences were found in richness when CI and CD were compared (p = 0.0017). Only six genera detected with the CD approach were not seen with the CI approach. Those genera were identified as unclassified Alcaligenaceae, *Alcaligenes*, unclassified Bacillales, unclassified bacteria, *Serratia*, and unclassified Staphylococcaceae. Also, no differences were found when the bacterial genera richness between organs salivary glands or gut was compared in this study (p = 0.97) (S3 Fig).

Although the Northern region showed a higher bacterial richness when it was compared to the Middle Magdalena region, there were no statistically significant differences (p = 0.06) (S3 Fig). Xu et al. (2015) had reported that microbial diversity in *R*. *microplus* gut might be influenced by the geographic location; however, their results were based on PCR and Denaturing Gradient Gel Electrophoresis without statistical analyses [11].

In this study, unclassified Alcaligenaceae, *Alcaligenes*, unclassified Bacillales, unclassified bacteria, *Serratia*, and unclassified Staphylococcaceae were identified using the culture-dependent approach. A previous study in ticks only reported possible contamination with *Staphylococcus sciuris* as a common finding in the skin of cattle and other animals [35]. Other work reported the frequent detection of *Staphylococcus* in bovine skin and *Methylobacterium* as an environmental contaminant [12]. However, *Staphylococcus sciuris* neither *Methylobacterium* were detected in this study.

Several authors have reported significant variation in bacterial abundance based on the degree of tick engorgement [12,36]. Therefore, a limit to the results in this study whit partially-fed female ticks is that it cannot be extrapolated to different nutritional status, sex or developmental stages, and the influence of such aspects to bacterial diversity associated with *R*. *microplus* should be subject to future studies.

**Fig 3:**
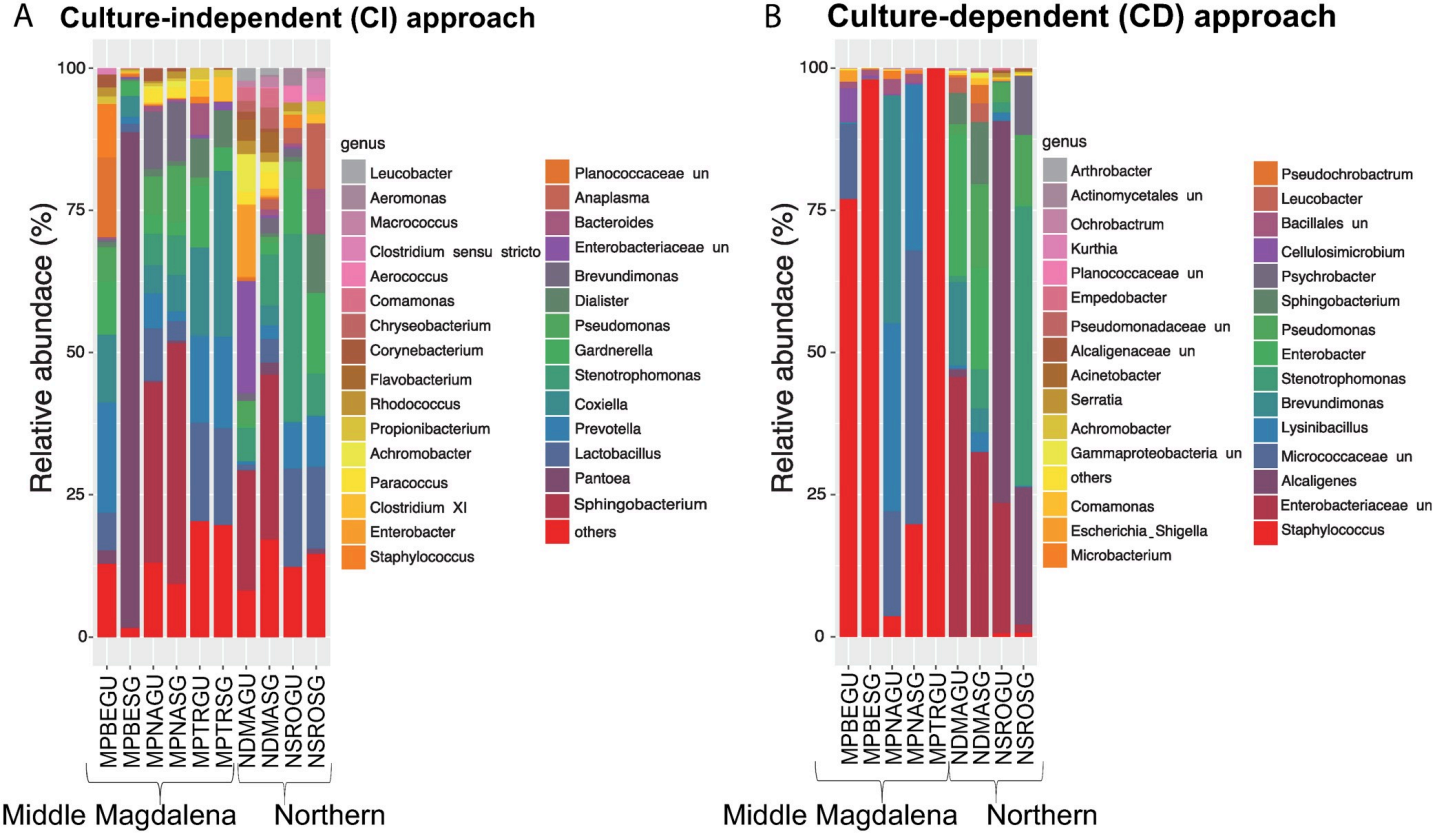
Relative abundance of bacteria at the genus level. (A) Samples obtained with the culture-independent (CI) approach, (B) culture-dependent (CD) approach. Data are presented in proportion (%) to all sequences classified at this taxonomy level. Key: M: Middle Magdalena region, N: Northern region; PBE: Puerto Berrío; PNA: Puerto Nare; PTR: Puerto Triunfo; DMA: Don Matías; SRO: Santa Rosa de Osos; GU: Gut; SG: Salivary glands; un: unclassified.

The most abundant genera detected with the CI approach were *Sphingobacterium* (12%), *Pantoea* (9%) and *Lactobacillus* (8%). *Staphylococcus* (< 1%) was less abundantly detected in all samples except two (Fig 3A). Regarding the CD approach, the most abundant genera were *Staphylococcus* (28%), unclassified Enterobacteriaceae (15%), *Alcaligenes* (15%), and unclassified Micrococcaceae (9%) (Fig 3B). These results are consistent with other research where *Staphylococcus* genus and several species of the Enterobacteriaceae family were found to predominate in the gut of *R*. *microplus* cattle ticks [5].

### Bacterial beta diversity

This analysis displayed statistical differences in the bacteria community between methodological approaches (CI) and (CD) (PERMANOVA: F-value = 3.4417; R2 = 0.16837; p < 0.001) and between salivary glands and gut (PERMANOVA: F-value = 2.3472; R2 = 0.12132; p = 0.004) the R2 values indicating that the covariates explained a large portion of variation between bacterial communities.

### The bacterial core of *R*. *microplus*

**Fig 4:**
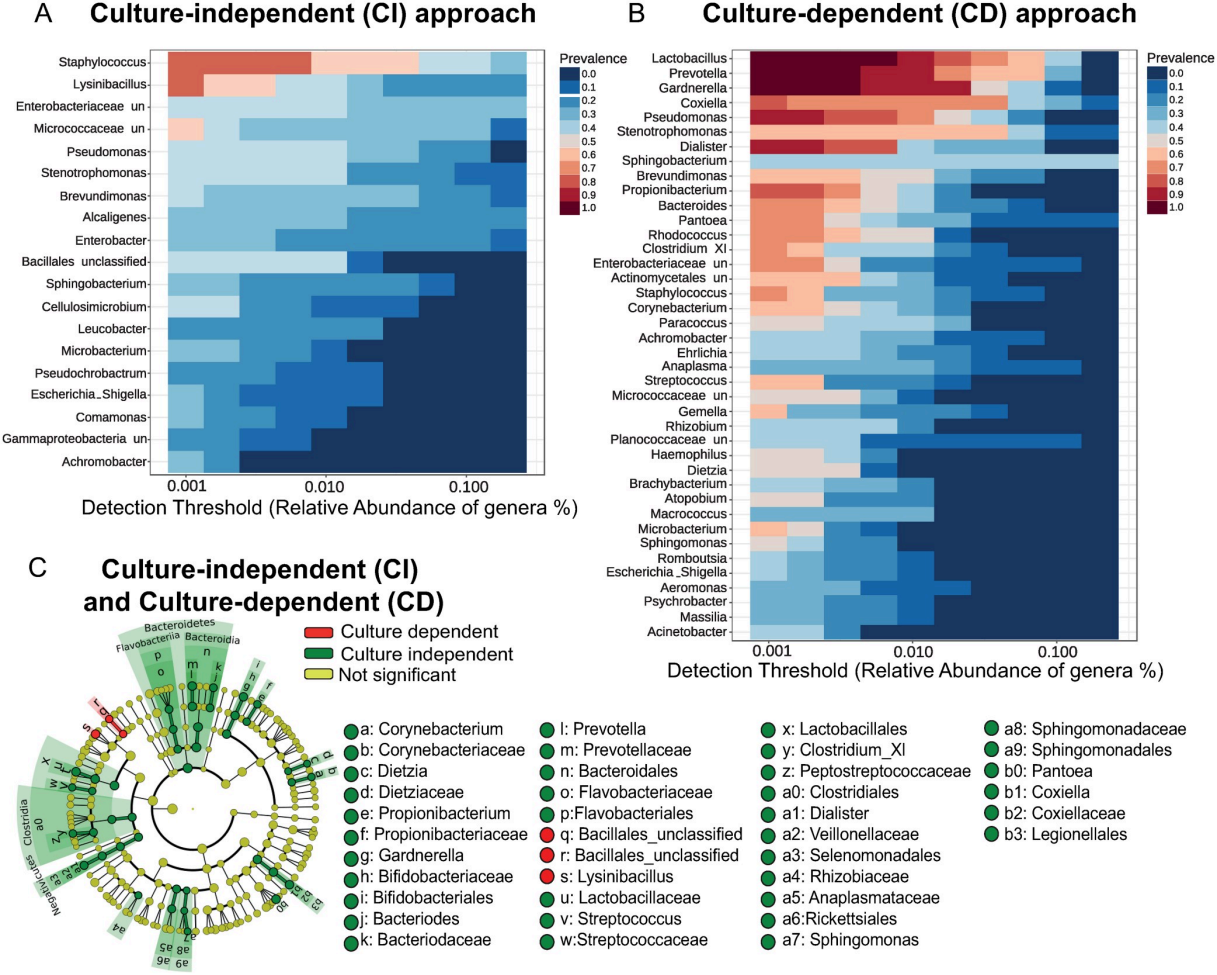
Comparative analysis of bacterial core community by 16S rRNA sequences obtained to samples of R. microplus using the CI and CD approaches. (A) Heatmap displays the genus-level core of the relative abundance of genera based on salivary glands and guts from *R*. *microplus* ticks from the Northern and the Middle Magdalena regions of Antioquia, data obtained using the CI approach. (B) Heatmap displays the genus-level core of the relative abundance of genera based on salivary glands and gut from *R*. *microplus* ticks from the Northern and the Middle Magdalena regions of Antioquia, data obtained using the CD approach; (%) The proportion and the color spectrum represent the abundance of each genus, dark blue represents minimum relative abundance, and dark red represents maximum relative abundance. (C) Biomarker taxa identified in *R*. *microplus* based on data comparison between the culture-independent (green) and the culture-dependent approaches (red), and non-significant (yellow).

**Fig 5:**
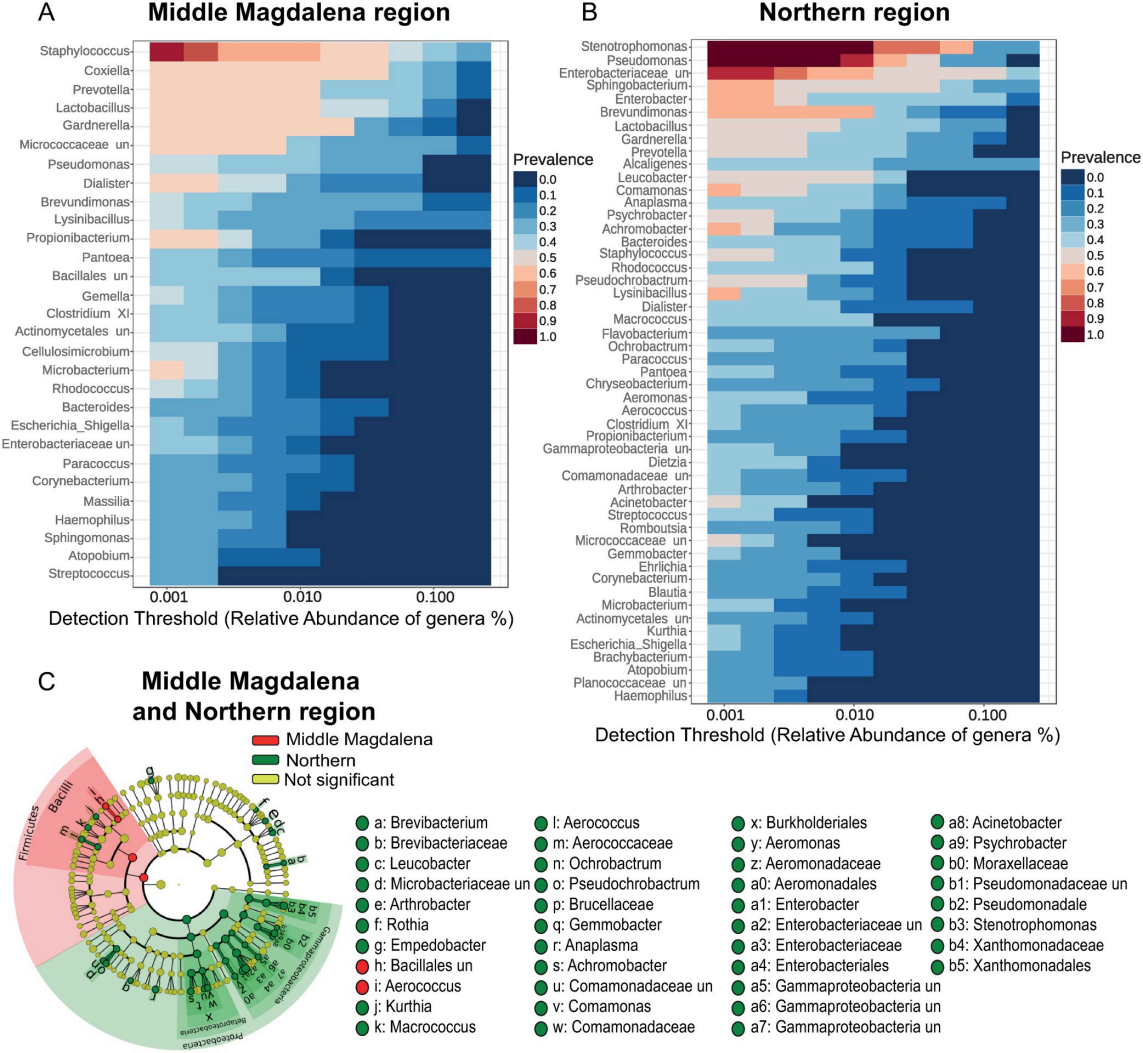
Comparative analysis of bacterial core community by 16S rRNA sequences obtained to samples of *R*. *microplus* from the Northern and the Middle Magdalena regions. (A) Heatmap displays the genus-level core of the relative abundance of genera based on salivary glands and guts from *R*. *microplus* ticks from the Middle Magdalena region, including data from both CI and CD approaches. (B) Heatmap displays the genus-level core of the relative abundance of genera based on salivary glands and guts from *R*. *microplus* ticks from the Northern region, including data with both CI and CD approaches (%). The proportion and the color spectrum represent the abundance of each genus, dark blue represents minimum relative abundance, and dark red represents maximum relative abundance. (C) Biomarker taxa identified *R*. *microplus* specimens from the Northern (green) and the Middle Magdalena (red) regions and non-significant (yellow).

The bacterial core community, regardless of the origin of the samples and the type of organ, showed the genera *Staphylococcus* and *Lysinibacillus*, and the family Enterobacteriaceae with the highest relative abundance in the CI approach (Fig 4A). *Lactobacillus*, *Prevotella*, *Gardenella*, and *Coxiella* were the four genera with the highest relative abundance in the CD approach (Fig 4B). On the other hand, *Staphylococcus*, *Coxiella*, and *Prevotella* were the three genera with the highest relative abundance in the samples from Middle Magdalena, regardless of the approach or the type of organ analyzed (Fig 5A). *Stenotrophomonas* and P*seudomonas* were the two genera with the highest relative abundance in the Northern region (Fig 5B).

A previous study reported *Coxiella* and *Bacillus* as the most prominent bacterial genera present in seabird ticks [37]. *Staphylococcus* has been previously reported in *R*. *microplus* [11] and almost all tick species. This observation might be explained by the fact that it is associated with the microbiota of the skin of mammalian hosts [38]. The genus *Pseudomonas* is considered ubiquitous in the environment and is frequently detected as one of the main genera in several tick species such as *I*. *scapularis* [36], *R*. *turanicus*, *R*. *sanguineus* [39], *A*. *americanum* [40] and in samples of salivary glands and gut of *A*. *maculatum* [41].

The increased abundance that environmentally acquired microbes such as *Bacillus*, *Pseudomonas*, and Enterobacteriaceae is associated with decreased pathogen colonization in ticks *Ixodes* [42].

In this study with the CD approach, the genus *Lysinibacillus* (order Bacillales) was identified. This genus has been reported in ticks *Dermacentor* [43], in *R*. *microplus* [5] and *I*. *scapularis* [44]. Furthermore, the entomopathogenic potential of *Lysinibacillus* species have been used to biological control of mosquito larvae [45]. This highlights the need to determine the role of tick endosymbionts.

### Biomarker analysis

The analysis of biomarkers in the CI approach identified several OTUs belonging to the phylum Bacteroidetes and Firmicutes. Regarding the former, classes Flavobacteriia and Bacteroidia were over-represented, and the latter, classes Clostridia and Negativicutes were also over-represented (Fig 4C, OTUs highlighted in green). With the CD approach, the genus *Lysinibacillus* (order Bacillales) was identified as a biomarker (Fig 4C, OTUs highlighted in red). It should be noted that with the CI approach, and the genus *Coxiella* was detected as a biomarker. This finding is not unexpected given that some members of this genus have demanding culture conditions or reproduce in an obligate intracellular way (Fig 4C). Biomarkers found in the CD samples must be interpreted with caution since optimization and culture media may affect their reproducibility, as it has been previously reported [11].

In the Middle Magdalena region, an over-representation of OTUs of the Firmicutes phylum and the Bacilli classes were detected (Fig 5C, OTUs highlighted in red). Proteobacteria phylum and the classes Gammaproteobacteria Betaproteobacteria were predominant in the Northern region (Fig 5C, OTUs highlighted in green). The microbial profiles might serve as biomarkers of infection frequency and transmission in ticks [46]. Therefore the bacterial diversity profiles observed in this study might provide an initial understanding of potential pathogen transmission by *R*. *microplus* in these areas.

### Analysis using sequences of the V3 through V4 regions of the16S rRNA gene

**Fig 6:**
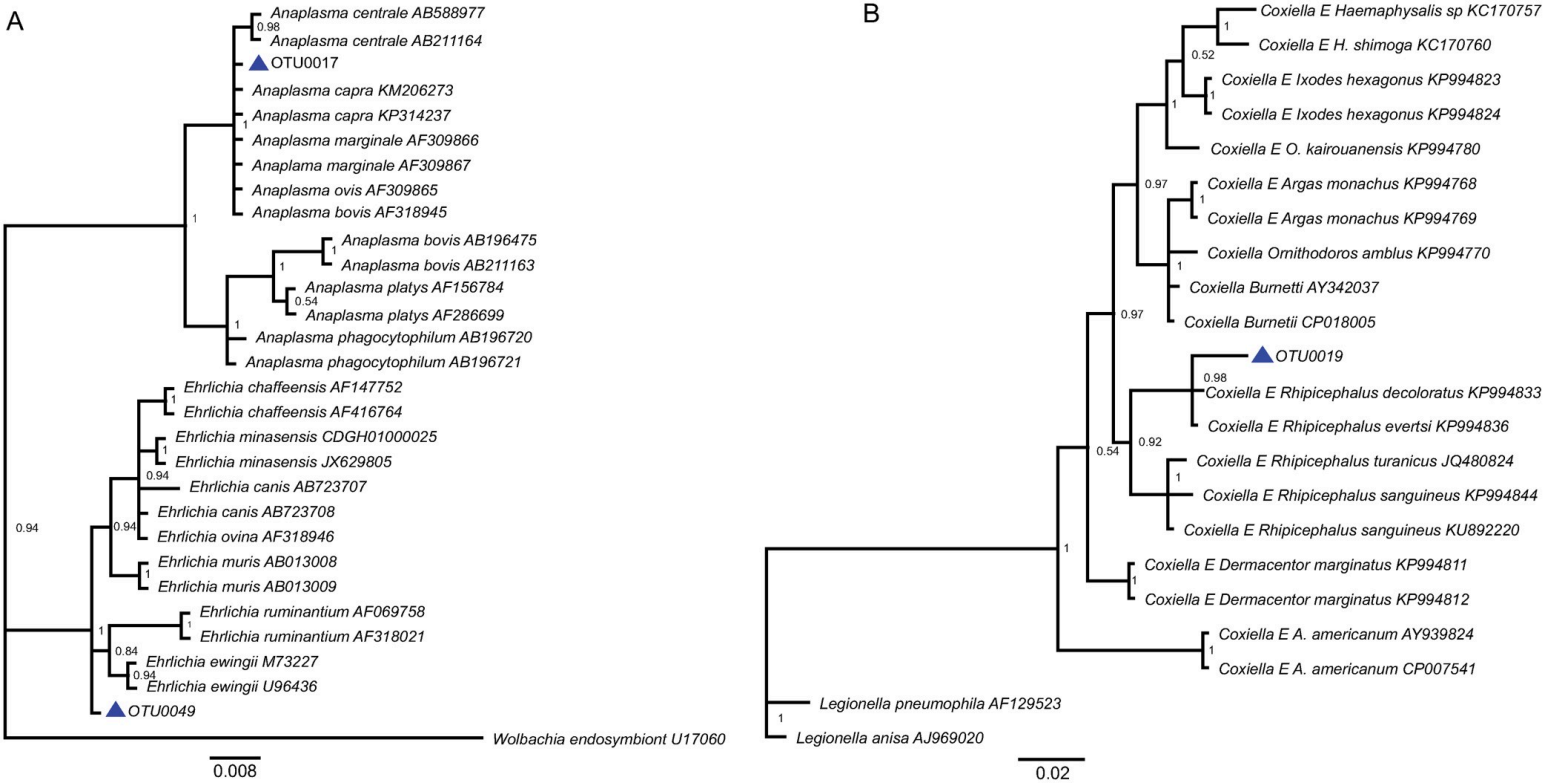
Molecular phylogeny analysis based on 16S rRNA. (A) Phylogenetic relationship of *Anaplasma* and *Ehrlichia* OTUs. Outgroup comprises *Wolbachia* endosymbiont of *Drosophila mauritiana*, (B) Phylogenetic relationship of *Coxiella* OTUs. Outgroup comprises *L*. *pneumophila* and *L*. *anisa*. Key: The triangle in blue correspond to the OTUs of this study.

In this study, the partial sequence of 16S rRNA corresponding to OTU0017 suggests the phylogenetic relationship of this OTU with different species of the genus *Anaplasma*; however, the definition of this relationship was not achieved at a species level. A similar result was observed with the sequence corresponding to the OTU0049 and the phylogeny analysis with reference sequences of the genus *Ehrlichia* (Fig 6A). A previous study that noted the relevance of *Anaplasma* as tick-borne bacteria, also suggested that midgut and salivary glands are specific barriers to efficient tick transmission [47]. Another study in Colombia found various species of *Anaplasma* and *Ehrlichia* circulating in different ticks species, including *R*. *microplus* from Cordoba (department to the North of Colombia) [48]. Regarding the phylogeny analysis of *Coxiella* sp., the OTU0019 was closely related to *Coxiella*-like endosymbionts (Fig 6B), previously found in *R*. *decoloratus* and *R*. *evertsi*, which have been reported as evolutionarily related but distinct to *C*. *burnetii* [49]. Because *Coxiella* species have been previously reported in ticks of the genera *Amblyomma*, *Dermacentor*, *Ixodes*, *Ornitodorus* and *Rhipicephalus* [39,50,51], it might be useful to use a multilocus sequence typing strategy in future investigations, in order to achieve the *Coxiella* identification in *R*. *microplus* [52].

In contrast to earlier findings, *Rickettsia*, which had been reported as one of the most frequent bacteria genera detected in *R*. *microplus* [12], *Ixodes scapularis* [53], *Amblyomma americanum* [54] and *Dermacentor occidentalis*, was not found in this study [55]. This is an important issue for future research, given the possibility of competition between *Coxiella* and *Rickettsia*, as it had been previously suggested in *A*. *americanum* [54]. Bacterial competition was presented for *I*. *scapularis* since it was found that the frequencies of the Rickettsiaceae family were inversely related to those of Pseudomonadaceae or Enterobacteriaceae [56]. Besides, error rates during sequencing and the effect of taxonomic resolution could interfere to some degree, hindering thus an accurate identification of bacteria genera [57]. A previous study proposed that the bioinformatic analysis may also result biased by the capability and resolution of the similarity algorithm employed [58].

Although in this study, the differences between cattle breeds were not evaluated, their influence on the microbiome of the *R*. *microplus* ticks of the two areas should be considered in future studies. Furthermore, employ other bacteria culture methodologies to permit the research of other genera that can be obtained through anaerobic bacteria culture and in the eukaryotic cell culture allowing the isolation and growth of obligate intracellular bacteria that can be transmitted by ticks such as *Coxiella*, *Ehrlichia*, *Anaplasma*, *Rickettsia*, and others.

## Conclusions

This work makes an initial description of bacterial diversity in salivary glands and gut of *R*. *microplus* adult female ticks from Northern and the Middle Magdalena regions from Antioquia, Colombia. This knowledge is essential because this specie considered the most predominant tick in livestock systems.

Through a complementary use of molecular procedures and traditional microbiological methods, a great diversity at the phylum and genus level was found in the *R*. *microplus* specimens analyzed. The culture-independent approach used in this study resulted in the detection of genera such as *Coxiella*, *Anaplasma*, and *Ehrlichia*, described as agents with public health importance. In addition to other endosymbionts, such as *Lysinibacillus*, previously reported as a potential tool for biological control, were detected for the first time in *R*. *microplus*. However, more studies are needed to improve this knowledge, their potential implications for tick-borne diseases, and endosymbionts related to *R*. *microplus* from South America ticks’ populations.

## Supporting information

S1 Table Data on *R*. *microplus* specimens analyzed, the number of reads, and OTUs obtained. (TIF)

S1 Fig Phylogeny-based Maximum Likelihood method and T92+G model using partial sequences of the mitochondrial 16S ribosomal RNA gene [59]. There were a total of 358 positions in the final dataset from tick’s specimens collected in this study from North and Middle Magdalena region of Colombia and sequences from the NCBI. The tree is drawn to scale, with branch lengths measured in the number of substitutions per site. This analysis involved 25 nucleotide sequences. Outgroup comprises *Ornithodoros rostratus*. Key: The triangle in blue correspond to the samples of this study. DMA: Don Matías; SRO: Santa Rosa de Osos to the North region, and PBE: Puerto Berrio; PNA: Puerto Nare; PTR: Puerto Triunfo to the Middle Magdalena región. The three-letter country codes correspond to the samples to other studies used the officially assigned ISO 3166–1 alpha-3 codes (https://www.iso.org/iso-3166-country-codes.html).
(TIF)

S2 Fig Rarefaction curves. (A) Samples analyzed from the culture-dependent (CD) approach, (B) culture-independent (CI) approach. Key: M: Middle Magdalena region, N: North region; PBE: Puerto Berrio; PNA: Puerto Nare; PTR: Puerto Triunfo; DMA: Don Matías; SRO: Santa Rosa de Osos; GU: Gut; SG: Salivary glands.
(TIF)

S3 Fig OTUs observed for Alpha diversity. (A) culture-dependent (CD) and culture-independent (CI), (B) Gut and Salivary glands, (C) Middle Magdalena and North region.
(TIF)

S4 Fig Diversity index of culture-dependent (CD) and culture-independent (CI) approaches. (A) Shannon phylum, (B) Simpson phylum, (C) Shannon genus, (D) Simpson genus.
(TIF)
