# Supplementary figures and images for: Assessment of bacterial diversity of Rhipicephalus microplus ticks from two livestock agroecosystems in Antioquia, Colombia
Source: PLoS One. 2020 Jul 1;15(7):e0234005. doi: 10.1371/journal.pone.0234005 (PMC7329104; doi:10.1371/journal.pone.0234005)

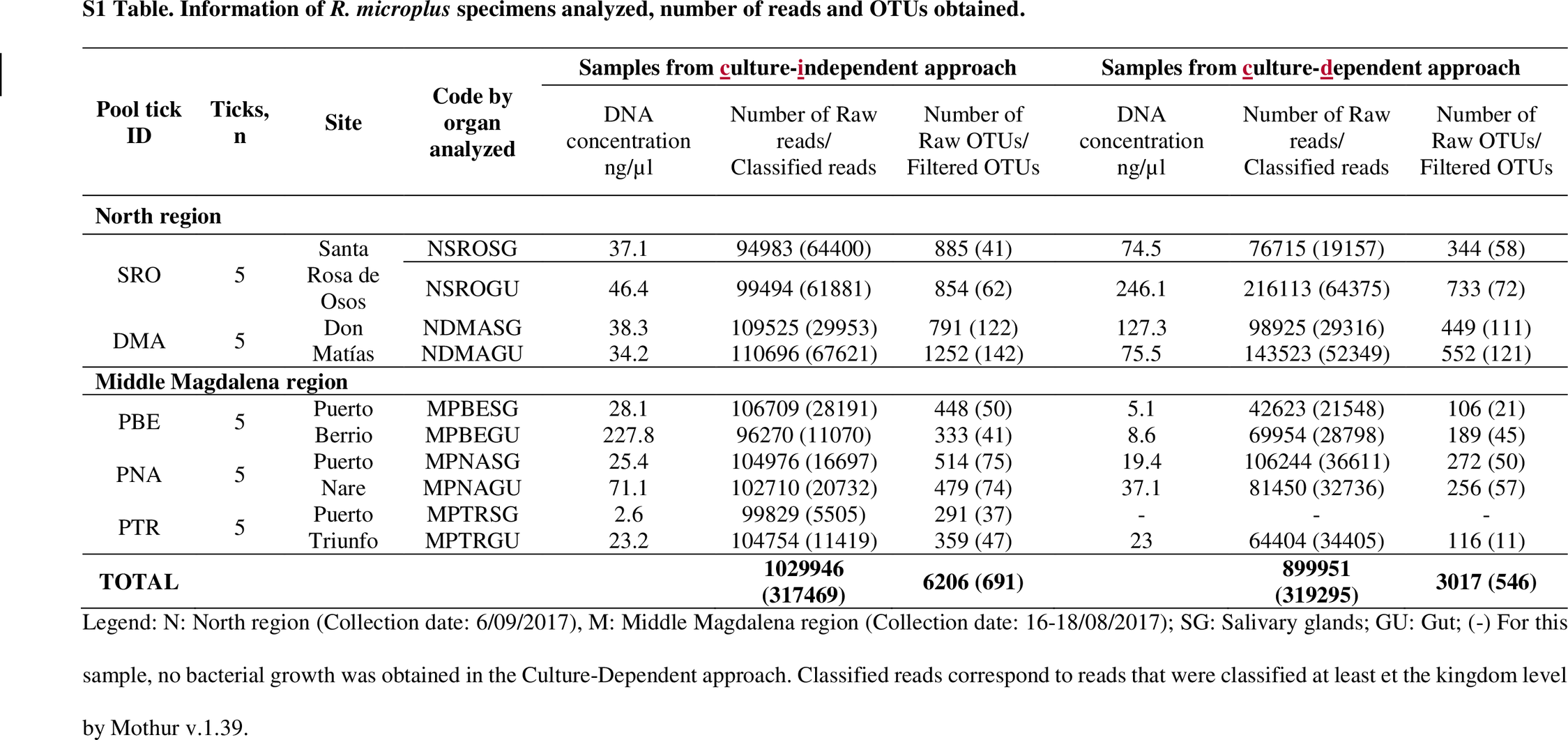

Supplement: S1 Table — (TIF) [file pone.0234005.s001.tif]

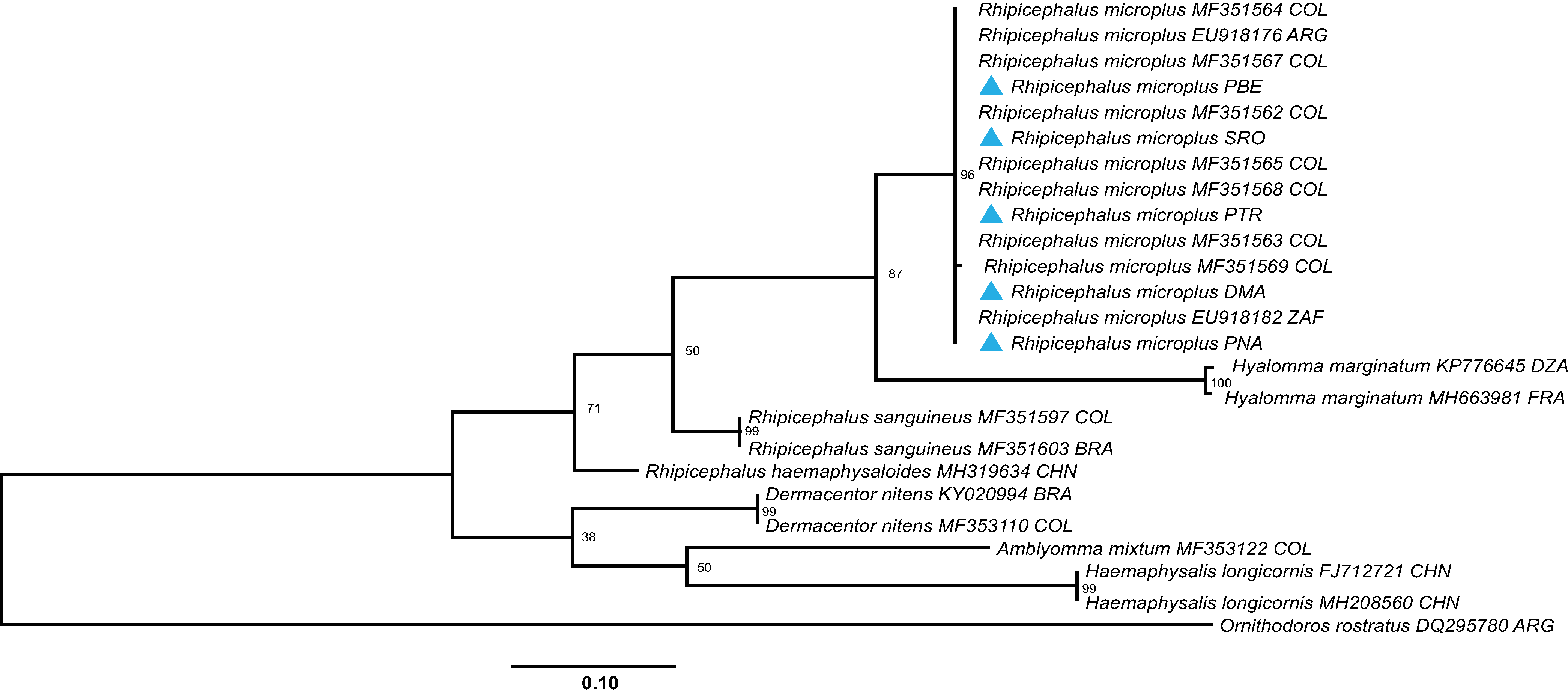

Supplement: S1 Fig — There were a total of 358 positions in the final dataset from tick’s specimens collected in this study from North and Middle Magdalena region of Colombia and sequences from the NCBI. The tree is drawn to scale, with branch lengths measured in the number of substitutions per site. This analysis involved 25 nucleotide sequences. Outgroup comprises Ornithodoros rostratus. Key: The triangle in blue correspond to the samples of this study. DMA: Don Matías; SRO: Santa Rosa de Osos to the North region, and PBE: Puerto Berrio; PNA: Puerto Nare; PTR: Puerto Triunfo to the Middle Magdalena región. The three-letter country codes correspond to the samples to other studies used the officially assigned ISO 3166–1 alpha-3 codes (https://www.iso.org/iso-3166-country-codes.html). (TIF) [file pone.0234005.s002.tif]

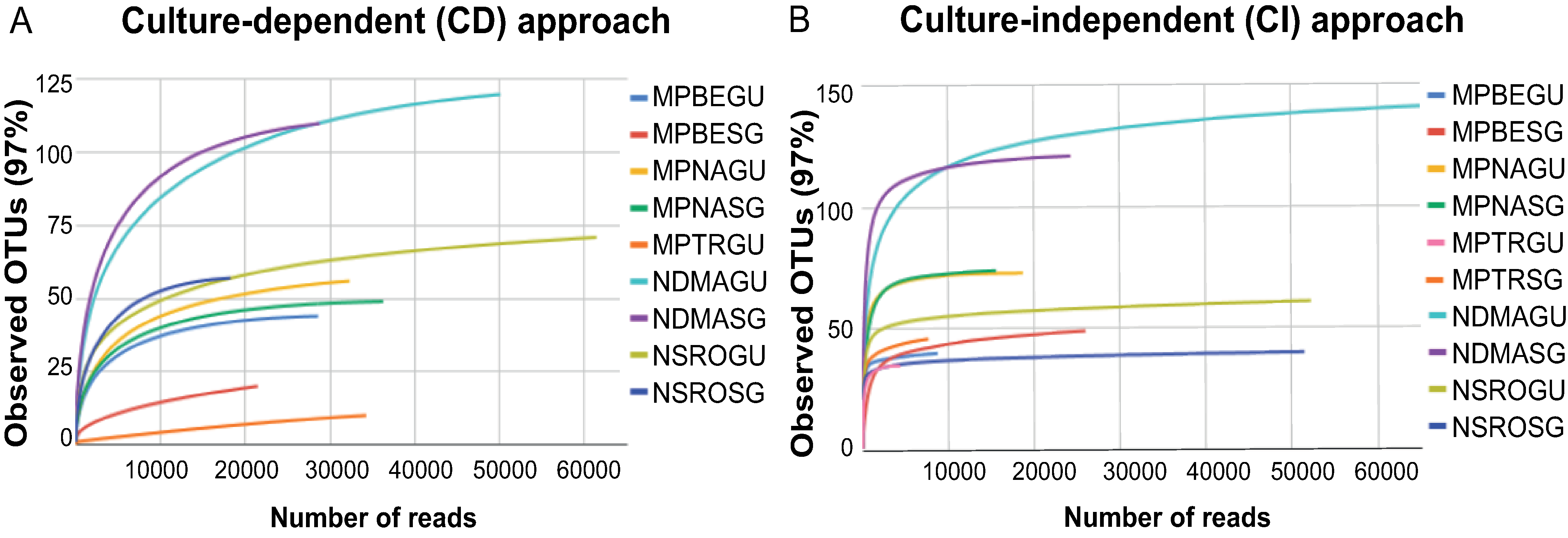

Supplement: S2 Fig — (A) Samples analyzed from the culture-dependent (CD) approach, (B) culture-independent (CI) approach. Key: M: Middle Magdalena region, N: North region; PBE: Puerto Berrio; PNA: Puerto Nare; PTR: Puerto Triunfo; DMA: Don Matías; SRO: Santa Rosa de Osos; GU: Gut; SG: Salivary glands. (TIF) [file pone.0234005.s003.tif]

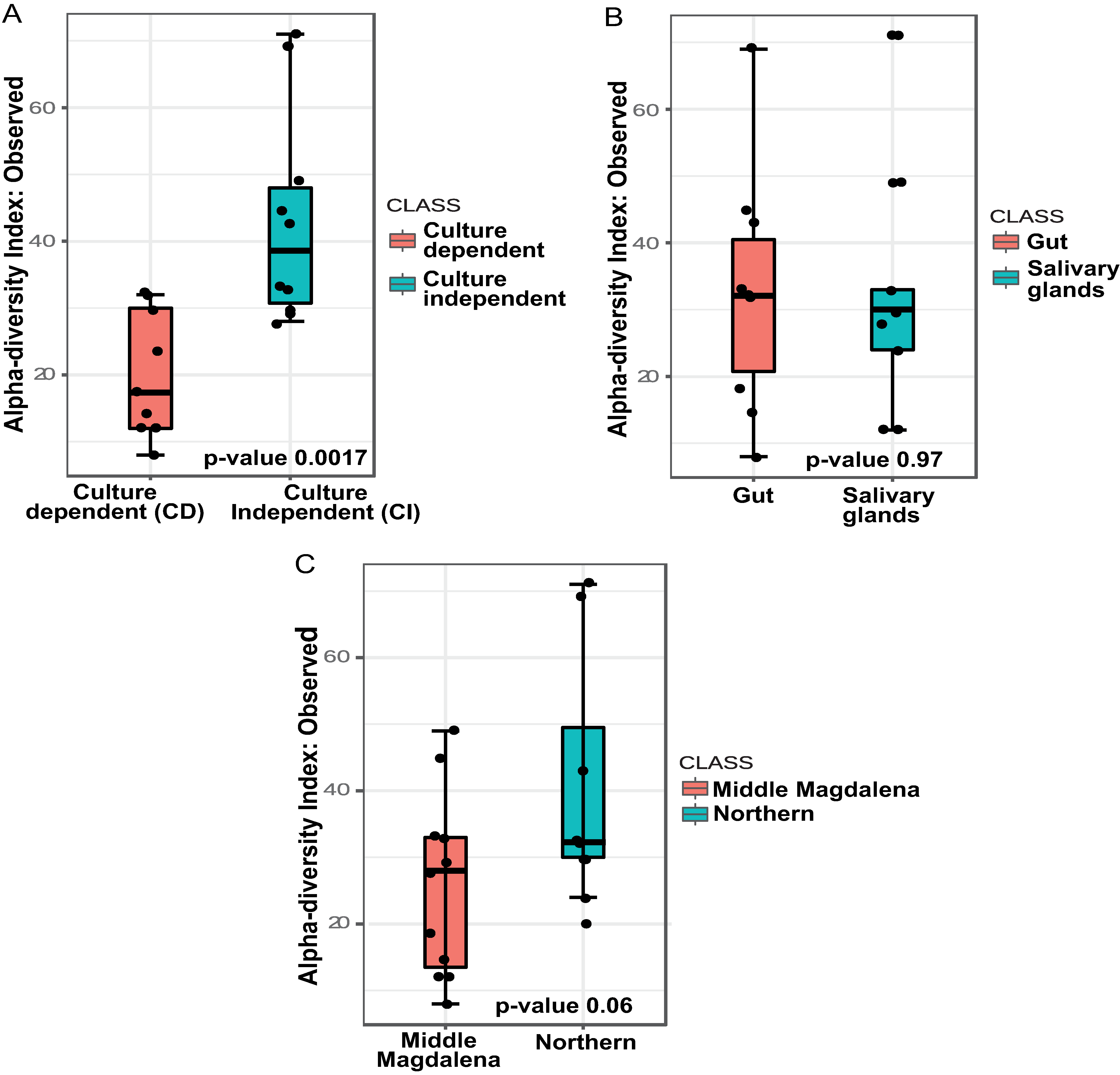

Supplement: S3 Fig — (A) culture-dependent (CD) and culture-independent (CI), (B) Gut and Salivary glands, (C) Middle Magdalena and North region. (TIF) [file pone.0234005.s004.tif]

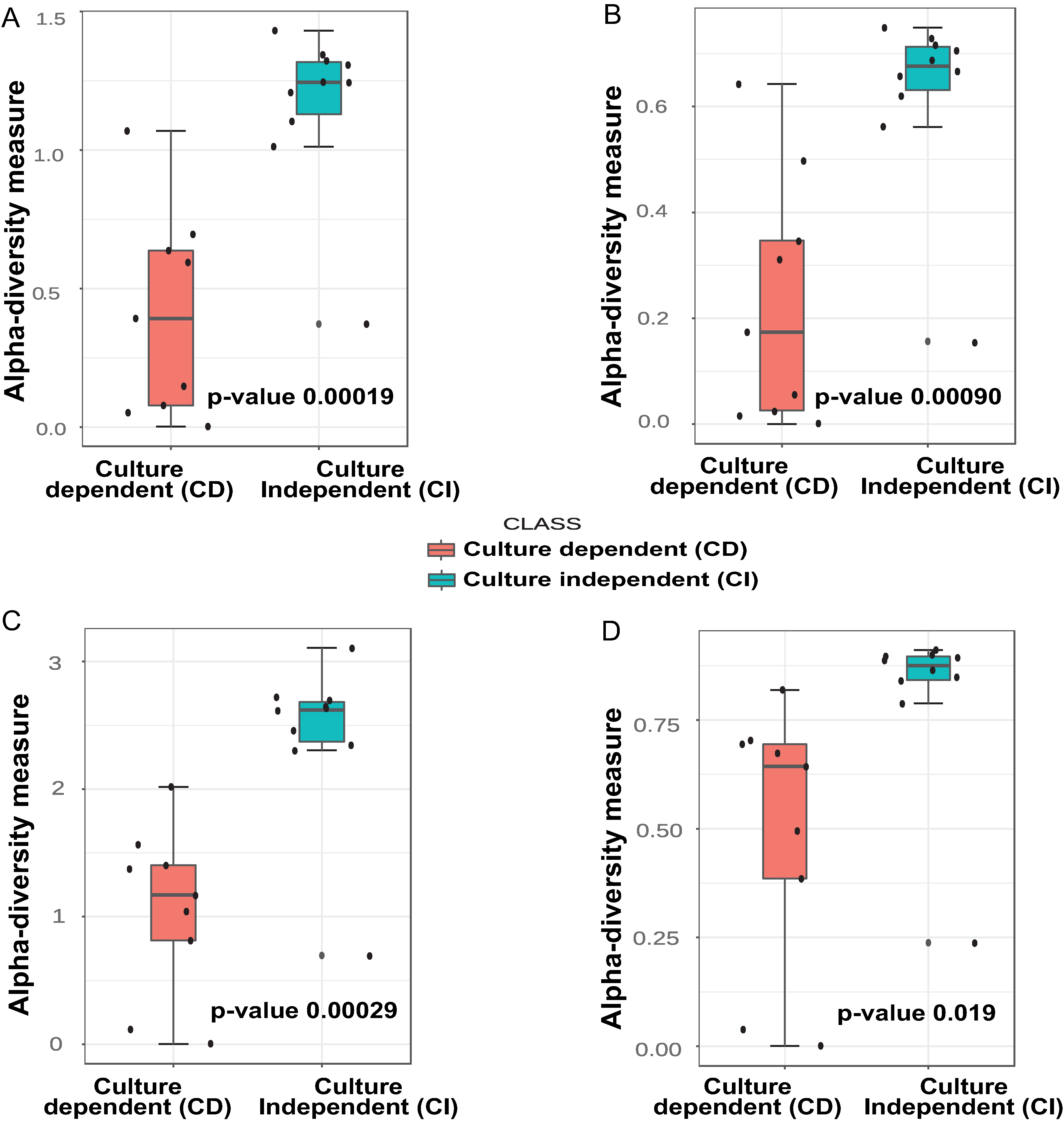

Supplement: S4 Fig — (A) Shannon phylum, (B) Simpson phylum, (C) Shannon genus, (D) Simpson genus. (TIF) [file pone.0234005.s005.tif]
